# Impact of Multiple COVID-19 Waves on Gynaecological Cancer Services in the UK

**DOI:** 10.3390/cancers15041273

**Published:** 2023-02-16

**Authors:** Samuel Oxley, Ashwin Kalra, Michail Sideris, Nicole Itzkowitz, Olivia Evans, Emma Christine Atakpa, Adam R. Brentnall, Nina Dworschak, Faiza Gaba, Rhian Gabe, Sudha Sundar, Nick Wood, Shibani Nicum, Alexandra Taylor, Stephen Dobbs, W. Glenn McCluggage, Andy Nordin, Rosa Legood, Sean Kehoe, Sadaf Ghaem-Maghami, Ranjit Manchanda

**Affiliations:** 1 Wolfson Institute of Population Health, Cancer Research UK, Barts Centre, Queen Mary University of London, Charterhouse Square, London EC1M 6BQ, UK; 2 Department of Gynaecological Oncology, Royal London Hospital, Barts Health NHS Trust, London E1 1BB, UK; 3 Institute of Applied Health Sciences, University of Aberdeen, Aberdeen AB24 3FX, UK; 4 Institute of Cancer and Genomic Sciences, University of Birmingham, Birmingham B15 2TT, UK; 5 Lancashire Teaching Hospitals NHS Foundation Trust, Preston PR2 9HT, UK; 6 Institute of Cancer Research, University College London, London WC1E 6DD, UK; 7 Royal Marsden NHS Foundation Trust, London SW3 6JJ, UK; 8 Belfast City Hospital, Belfast Health and Social Care Trust, Belfast BT9 7AB, UK; 9 Department of Pathology, Belfast Health and Social Care Trust, Belfast BT12 6BA, UK; 10 East Kent Gynaecological Oncology Centre, Queen Elizabeth the Queen Mother Hospital, Margate CT9 4AN, UK; 11 Department of Health Services Research and Policy, London School of Hygiene & Tropical Medicine, London WC1H 9SH, UK; 12 Faculty of Medicine, Department of Surgery & Cancer, Imperial College London, London SW7 2AZ, UK; 13 MRC Clinical Trials Unit at UCL, Institute of Clinical Trials & Methodology, Faculty of Population Health Sciences, University College London, London WC1V 6LJ, UK; 14 Department of Gynaecology, All India Institute of Medical Sciences, New Delhi 110029, India

**Keywords:** COVID-19, multidisciplinary team, gynaecological cancer

## Abstract

**Simple Summary:**

This study aimed to assess the impact of multiple COVID-19 waves on gynaecological cancer services across the UK. A survey was sent to staff in UK cancer hospitals after the first wave in 2020, and this was repeated for the second and third waves in 2021 and 2022. This showed that referrals halved during the first wave, and half of hospitals reported reduced staffing. The number of operations performed substantially fell, with many being postponed. Many hospital meetings and appointments were conducted by videoconferencing or telephone rather than in-person. By the second wave, referrals were at normal levels, with fewer reductions in staffing, operations, and other services. By the third wave, there were worse staffing reductions, similar to 2020, despite normal workloads. Our analysis shows the major impact of COVID-19 on gynaecological cancer services, highlights serious staffing shortages, and gives insights into the adaptations needed in the future.

**Abstract:**

Background: This study aimed to assess the impact of multiple COVID-19 waves on UK gynaecological-oncology services. Methods: An online survey was distributed to all UK-British-Gynaecological-Cancer-Society members during three COVID-19 waves from 2020 to2022. Results: In total, 51 hospitals (including 32 cancer centres) responded to Survey 1, 42 hospitals (29 centres) to Survey 2, and 39 hospitals (30 centres) to Survey 3. During the first wave, urgent referrals reportedly fell by a median of 50% (IQR = 25–70%). In total, 49% hospitals reported reduced staffing, and the greatest was noted for trainee doctors, by a median of 40%. Theatre capacity was reduced by a median of 40%. A median of 30% of planned operations was postponed. Multidisciplinary meetings were completely virtual in 39% and mixed in 65% of the total. A median of 75% of outpatient consultations were remote. By the second wave, fewer hospitals reported staffing reductions, and there was a return to pre-pandemic urgent referrals and multidisciplinary workloads. Theatre capacity was reduced by a median of 10%, with 5% of operations postponed. The third wave demonstrated worsening staff reductions similar to Wave 1, primarily from sickness. Pre-pandemic levels of urgent referrals/workload continued, with little reduction in surgical capacity. Conclusion: COVID-19 led to a significant disruption of gynaecological-cancer care across the UK, including reduced staffing, urgent referrals, theatre capacity, and working practice changes. Whilst disruption eased and referrals/workloads returned to normal, significant staff shortages remained in 2022, highlighting persistent capacity constraints.

## 1. Introduction

The UK experienced significant morbidity and mortality, and a disruption to daily life as a result of COVID-19 during 2020–2022. The first national lockdown occurred on 23 March 2020, and continued in its most severe form until 1 June 2020 (the first wave), although major restrictions remained [1]. A more transmissible and deadly UK-specific variant (B.1.1.7, WHO name Alpha) [2] was identified in September 2020, and triggered a sharp rise in hospital admissions and deaths, culminating in a second national lockdown during December 2020–April 2021 (second wave), with regional variations during this period [3]. Measures were relaxed following a reduction in hospital admissions and deaths, until the identification of another more transmissible variant in November 2021 (B.1.1.529, Omicron) [4]. This resulted in a further peak in hospital admissions during December 2021–March 2022 (third wave), with an associated tightening of restrictions, although with a lower mortality than in previous waves due in part to widespread vaccination [5].

COVID-19 affected all areas of healthcare including hospital-based gynaecological cancer care [6]. Elective and emergency activities were reduced due to reallocation of staff and resources towards COVID-19-related care, and the health system had to cope with additional stresses including staff sickness and self-isolation, reduced operating theatre availability, and supply chain shortages (including personal protective equipment) [6]. The majority of gynaecological cancer patients are older, often with co-morbidities rendering many at high risk for COVID-19. COVID-19 was associated with higher morbidity rates, the need for intensive care, and mortality in cancer patients [7]. Cancer services, therefore, were needed to balance the need for oncological treatment against the risks of contracting COVID-19 in healthcare settings.

UK gynaecological cancer services are organised into regional cancer networks in a hub-and-spoke model with 46 tertiary referral “centres” (cancer centres) surrounded by a total of 142 local district hospital “units” (cancer units) [8]. Each cancer centre is linked to a distinct set of units depending on region to provide gynaecological cancer care across that region. Units employ gynaecologists with a specialty interest in gynaecological cancer care. They provide diagnostic services and some restricted treatment of localised good prognostic disease such as FIGO stage IA Grade 1/2 endometrial cancers. The cancer centres provide subspecialist advice and the broad spectrum of treatment of all gynaecological cancer malignancies and oversight as well as coordination of management of all cancer cases, which occurs through a cancer multidisciplinary team (MDT) meeting. Cancer units are represented at the weekly cancer centre MDT meeting. All cancer cases must be discussed at a cancer centre MDT meeting, consisting of gynaecologists, radiologists, pathologists, clinical nurse specialists, gynaecological oncologists, medical oncologists, and radiation oncologists. Cancer units also host local MDT meetings, comprising gynaecologists, radiologists, pathologists, and clinical nurse specialists.

The National Health Service England communique 01559 (April 2020, v1) provided guidance on the management of cancer surgery and underlined the importance that cancer diagnoses, essential treatment, and urgent care must continue [9]. The British Gynaecological Cancer Society (BGCS) developed a framework for cancer centres (tertiary hospitals) and units (local district hospitals) to aid management decisions and provided a harms review template [10]. This identified patients where therapy may be delayed until the pandemic was controlled. The National Institute for Health and Care Excellence provided rapid guidance and principles for delivery of radiotherapy [11] and systemic anti-cancer treatments [12]. Mitigation strategies led to changes in surgical and chemotherapy plans, treatment delays, and introduction of regimens such as hypo-fractionated radiotherapy.

It is important to capture and analyse the significant service reconfiguration and changes to cancer care in order to understand their impact, identify the best strategies for future communicable disease epidemics/public health crises, and adaptations worth preserving. No study has reported the impact of COVID-19 on all UK-wide gynaecological cancer services. The UK COVID and Gynaecological Cancer Study (UKCOGS) was established in March 2020, with the aim of assessing the impact of COVID-19 on UK gynaecological oncology services. This forms the first part of this study, which aims to report the logistic and structural changes in UK cancer hospitals across three waves of COVID-19 in 2020, early 2021, and early 2022.

## 2. Materials and Methods

A UK-wide survey (File S1, Table S1: COVID-19 Survey of Gynae-oncology Centres and Units) was developed and peer-reviewed by BGCS officers consisting predominantly of gynaecological oncologists, along with some medical and radiation oncologists, representing all UK regions. The surveys captured data on the service provider (cancer centre or unit); changes to staffing; changes to MDT meeting functioning including frequency and attendance; virtual working; reduction in theatre time/surgical capacity; provision of minimal access surgery; reduction in medical and radiation oncology capacity; reduction in MDT workload and urgent/suspected cancer referrals (called ‘rapid-access’ referrals, for which UK guidelines mandate clinical review within 2 weeks); and relocation of activity to COVID-19-free sites.

The survey was hosted using Online Surveys (Jisc, Bristol, UK) and distributed electronically via email and BGCS member forums to all BGCS members in the UK. The BGCS comprises 238 consultant gynaecological oncologists from every cancer centre, 28 consultant radiation oncologists, 29 consultant clinical oncologists, 58 consultant gynaecologists based in cancer units, 94 clinical nurse specialists, and others including doctors in training. Any BGCS member was eligible to complete the survey if they had a good understanding of staffing and service provisions within their department; this would normally be the Head of Department or Clinical Director.

The survey was distributed on 29 April 2020, during the first wave of the pandemic (from March 2020) and remained open for return of responses until 15 May 2020. The same survey with minor changes (relating primarily to dates covered) was sent again to all BGCS members on 26 March 2021, to ask about the second wave from December 2020, and remained open until 30 April 2021. The survey was then sent a third time to all BGCS members on 26 February 2022 to ask about the third wave beginning December 2021 and remained open until 12 March 2022.

Responses to questions were summarised in total and by cancer centre or unit subgroups for each wave. Where there were multiple responders per site with discrepancies, we used responses that affirmed an impact on care, as some participating staff may not be aware of all changes (e.g., for other specialities than their own), and it is unlikely that respondents would indicate reductions without knowledge. A sensitivity analysis was performed when the negative response was used instead. For multiple responses per site for percentage reduction, we used the mean from all. We explored the potential association of crude rates for COVID-19 cases (at time of the survey) from English local authorities with structural changes to staff numbers. An ad-hoc Mann–Whitney two-sample test was used without adjustment for multiple comparisons to aid in the interpretation of one potential difference between centres and units. Analyses were undertaken in R (R Core Team, Vienna, Austria) [13].

### 2.1. Patient Public Involvement

We undertook extensive stakeholder engagement before commencing UKCOGS. We received support from the BGCS, the Royal College of Obstetricians and Gynaecologists, the National Cancer Research Institute Gynaecological Cancer Clinical Studies Group, and the British Association of Gynaecological Pathologists, along with endorsement from multiple charities and patient support groups such as Ovacome, Eve Appeal, Target Ovarian Cancer, Ovarian Cancer Action, Jo’s Cervical Cancer Trust, and GO Girls.

### 2.2. Ethics

This study was reviewed by the Barts Health Clinical Effectiveness Unit and by the Joint Research Management Office and Research & Development Team at Barts Health and the Queen Mary University of London (Project ID: 11123), and the COVID-19 committee. This was classified as a clinical audit, and formal ethics approval was not deemed necessary. The web-based survey was distributed by the BGCS, the clinicians completed it with implicit electronic consent, and the responders remained anonymous.

## 3. Results

**Figure 1:**
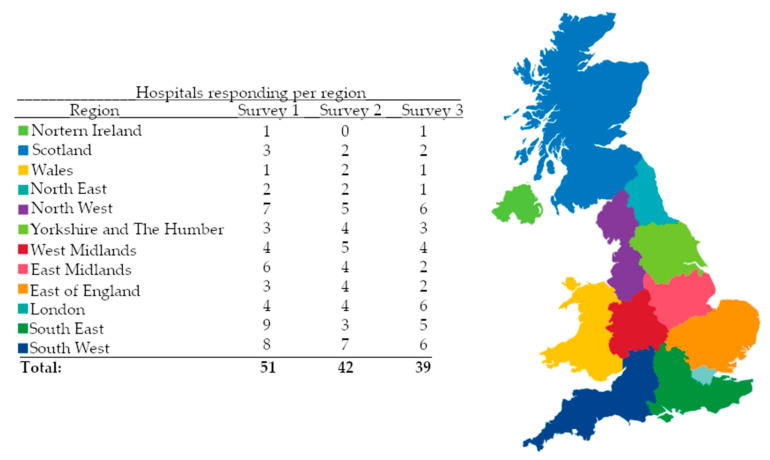
Number of hospitals responding to each survey by region of the United Kingdom.

A total of 51 hospitals responded to survey 1, for a total of 32/46 (70%) cancer centres and 19/142 units (13%), covering all UK regions. Ten centres and one unit had more than one responder for their site. In total, 42 hospitals responded to survey 2, for a total of 29/46 (63%) centres and 13/142 (9%) units. Three centres had more than one response. In total, 39 hospitals responded to survey 3, for a total of 30/46 (65%) centres and 9/142 (6%) units. Seven sites had two responders. The number of sites by region that responded are shown in Figure 1.

### 3.1. Staffing

Half (49%, 25/51) of the responding hospitals [53% (17/32) of cancer centres and 42% (8/19) of units] reported a reduction in staff numbers during Wave 1 (Table 1). In sites reporting a reduction in non-subspecialty trainee doctors, staffing was reduced by a median of 40% (IQR 25–100%), due to COVID-19-related sickness in 56% (14/25) and redeployment in 56% (14/25) of the total. One third (32%, 8/25) of hospitals reporting staff reductions had reduced numbers of gynae-oncology subspecialty trainees, with 24% (6/25) due to redeployment and 8% (2/25) due to sickness. Three quarters (76%, 19/25) of hospitals with staff reductions reported a loss of consultant staff: a median reduction of 28% (IQR 20–38%), with 68% (17/25) due to sickness and 8% (2/25) due to redeployment. A total of 61% (16/25) of hospitals reporting staff reductions saw a decrease in clinical nurse specialists by a median of 30% (IQR 20–50%), 36% (9/25) due to sickness, and 56% (13/25) due to redeployment being greater in the units.

Wave 2 saw a partial improvement. In total, 17% (7/42) of hospitals reported reduced staff numbers, as did 17% (7/29) of centres, and 0% (0/14) of units. Within hospitals reporting reductions, non-subspecialty trainee doctor and clinical nurse specialist shortages were caused equally by sickness and redeployment; however, consultant and subspecialty trainee shortages were only from sickness.

Wave 3 revealed worsening staff reductions as 44% (17/39) of hospitals reported reductions, mostly from sickness rather than redeployment for all groups.

No clear association was found between crude rates of COVID-19 by relevant local authorities and structural changes to staffing. The sensitivity analysis using negative responses where there had been discrepancy sites showed no material change to the findings (data not shown).

### 3.2. Gynaecological Cancer Referrals, MDT Workload, and Meetings

During the first wave, urgent/rapid access referrals fell by a median of 50% (IQR 25–70%), and weekly MDT meeting workloads fell by a median of 22% (2–48%) (Table 2), which was similar across centres and units. Waves 2–3 showed almost no reduction in urgent/rapid access referrals or overall workload compared to pre-pandemic levels.

All centres and units reported changes to MDT meeting functioning during Wave 1, from face-to-face pre-COVID-19, to virtual (using videoconferencing or telephone facilities), being completely virtual in 39% (20/51), and mixed virtual/face-to-face in 65% (33/51) of the total. A total of 41% (21/51) of hospitals reported reduced attendance, with a median 40% decrease. However, no hospital suspended or reduced the frequency of meetings.

The majority of hospitals (83%, 35/42) reported these adaptations continuing during Wave 2. A similar proportion (38%, 16/42) conducted these meetings virtually, with half conducting mixed virtual/face-to-face meetings (50%, 21/42). Attendance was reduced in 16% (7/43) of hospitals, with a median 25% decrease (IQR 25–35%). These changes persisted in Wave 3 with 85% (33/39) of hospitals reporting adaptations, such as virtual meetings in 44% (17/39) and mixed virtual/face-to-face meetings in 46% (18/39) of the total. In total, 13% (5/39) of hospitals reported reduced attendance compared to pre-COVID levels.

### 3.3. Virtual Clinics

A major shift towards virtual clinic appointments was reported during Wave 1, with remote consultations undertaken at a median of 75% (50–88%) of the time, across both centres and units (Table 2). Wave 2 revealed remote consultations becoming less common with a median of 25% (5–35%) of appointments, although one centre still reported rates of 71 -80%. Wave 3 showed a further reduction in remote consultations with a median of 0% (0–25%).

### 3.4. Service Provision

During the first wave, there was a median 40% (IQR 20–70%) reduction in theatre capacity. This was greater in units (60%, 32–88%) than in centres (30%, 12–55%)(*p* = 0.023) (Table 2). In total, 30% (IQR 16–57%) of planned surgeries were postponed, in both centres and units. The second wave had less reduction in theatre time (10%, 10–25%), with the impact being similarly greater in units (15%, 0–35%) than in centres (5%, 0–25%). Only 5% (0–15%) of surgical cases were postponed, which was similar across units and centres. By Wave 3, there was generally little reduction in theatre time (0%, 0–5%), although it was greater in units (0% (0–30%)), with one unit reporting an 80–90% reduction. A total of 5% (0–5%) of surgical cases was postponed.

Minimal access surgery provision was reduced during the first wave, being conducted in 86% (19/22) of centres and 58% (11/19) of units. This improved to baseline by the second wave, with 28/29 centres and 14/14 units performing this as they did pre-pandemic (one centre did not answer). All hospitals performed minimal access surgery in Wave 3.

Results from Wave 2 showed that 44% of sites required 13–14 days isolation for patients prior to major procedures, and 35% of sites required 3–4 days. By Wave 3, 26% (10/39) required 7 days isolation and 56% (22/39) required 3 days. All sites required a negative COVID-19 swab prior to any procedure, with 9% requiring two. A total of 14% of centres and units required patients to be vaccinated prior to major procedures, and 9% before even minor procedures.

No major reduction in non-surgical (medical oncology, clinical oncology, radiology, pathology and palliative care) access/capacity was reported in Wave 1. In Wave 2, one centre reported a 41–50% reduction in medical oncology access/capacity, and one centre and one unit reported a 31–40% reduction in clinical oncology access/capacity, with one centre reporting an 81–90% reduction in inpatient brachytherapy. This indicates a severe impact for a small number of hospitals; however, a large proportion of centres did not answer these questions. Reductions in non-surgical capacity were almost completely resolved by Wave 3.

A total of 56% (23/41) of hospitals moved operating off-site to the independent sector during the first wave, which reduced to 40% (17/43) by Wave 2. In Wave 1, central co-ordinating hubs were used to book surgical cases in 38% (15/39) of hospitals, and a similar proportion was seen in Waves 2 to 3 (Table 2). During Wave 2, one unit indicated that pathology was reported by another team when operating at a different location, while for all 41 other hospitals, pathology was reported by their usual in-house team. In total, 19% (8/42) of responding sites worked in a COVID-19-free hospital during Wave 2, and 67% (28/42) worked within COVID-19-free “green” zones within a non-COVID-19-free hospital. By Wave 3, fewer (10% (4/39)) hospitals operated off-site.

## 4. Discussion

### 4.1. Main Findings

Our study helps to quantify the major impact COVID-19 had on UK-wide gynaecological cancer service provision. Amongst reporting hospitals, urgent (rapid access) referrals approximately halved during Wave 1, but returned to pre-pandemic levels by Wave 2. Half of the reporting hospitals reported a median 40% staffing reduction during Wave 1, predominantly from COVID-19-related sickness amongst consultants, and redeployment of doctors in training. Whilst staff absences decreased during Wave 2, they worsened in Wave 3, predominantly from sickness/isolation, despite the return of high workloads. Theatre capacity reduced by 40% during Wave 1, with one third of operations postponed. Although fewer units responded than centres did, those that responded were less likely to undertake minimal access surgery during this period and experienced a greater surgical capacity reduction than the cancer centres did. This picture substantially improved by the second and third waves. Adaptations to working were rapidly introduced in 2020, with the majority of hospitals incorporating virtual MDT meetings and maintaining these arrangements into 2021. A majority of outpatient workload became virtual during Wave 1, although these rates fell during Waves 2 to 3. While overall figures showed reassuring improvements in many areas during Waves 2 to 3, several hospitals still experienced high ongoing disruption from substantial reductions in staffing or service provision, demonstrating an uneven impact across the country by geography and hospital type.

### 4.2. Strengths and Limitations

This is the only analysis on UK gynaecological cancer services during this period of repeated COVID-19 waves. Strengths include the capturing of data from the majority of cancer centres and from hospitals across all geographical regions in the UK, the reporting of many aspects of service provision, and the timing, covering three waves of the pandemic. The repeated surveying enables a comparison across years, showing where adaptations have been made and persisted as well as highlighting continued capacity issues where they exist. Limitations include a lower representation of cancer units, and potential selection bias in those who responded. We are unable to provide a breakdown of those completing the survey by speciality, although the majority of BGCS members are subspecialist surgical gynaecological oncologists. Respondents were therefore more likely to be surgeons in cancer centres and may have more limited knowledge of non-surgical treatments. We did not ask for any personal identifiers in order to encourage the unbiased and honest sharing of information. Few fields were left unknown, likely due to those completing the questionnaire not knowing about all areas related to gynaecological cancer care in their hospital. Nevertheless, those filling the questionnaire had a good understanding or overview of the relevant staffing/capacity/workload/structural issues in their department and were often the clinical lead or Clinical Director.

### 4.3. Comparison with Other Literature

This is the first and only national survey data on UK gynaecological cancer services during the COVID-19 pandemic and highlights changes over multiple COVID-19 waves. Other studies have reported on the impact on gynaecological oncology services at a single time point only early in the pandemic, with broadly similar adaptations seen in Germany [14], India [15], and Italy [16], and a less severe reduction in surgical capacity in Japan [17]. The reduction in cancer referrals during the first wave is documented internationally [18] and in the UK, with a reduction in cervical cancer diagnoses in northern England by 25% [19], and referrals of all cancer types from primary care [20]. Data from the UK National Radiotherapy Dataset show a 30% reduction in attendances for radiotherapy including for cervical cancer during the first wave, with a rapid increase in hypo-fractionated regimens [21]. The CovidSurg group found that 20.7% of surgical gynaecological oncology patients across 52 countries experienced a change in management including significant delay or cancellation, leading to resultant disease progression or death for many [22]. These findings are consistent with our results, although the level of detail in our survey allows for greater insights into service changes and the comparison across three years. Our data showed redeployment of staff including doctors in training. This is supported by an international survey of gynaecological oncology trainees, highlighting reduced surgical training in 50% of them, increased distress, particularly for trainees in accredited programmes, increased anxiety and depression in those off-sick, and additional time expected for completion of training by over 30% [23]. Other staff surveys have demonstrated high levels of burnout and low professional fulfilment in gynaecological oncology staff across two waves of COVID-19 in the USA [24], and more widely amongst oncologists in Europe [25].

### 4.4. Implications

We are able to correlate and assess the relative impact of COVID-19 on staffing and patient referrals/MDT workloads over time. Whilst staffing reductions were similar to workload reductions in Wave 1, this ratio worsened in Wave 2 (due to a return to normal workloads with reduced staffing), and substantially worsened in Wave 3 (due to severe staffing shortages with continuation of normal workloads). A major challenge for services in planning the recovery from COVID-19 is the impact seen on staff and safe staffing levels. The repeated severe shortages reported in our surveys, coupled with a return to pre-pandemic workloads, have applied and sustained high pressure on all staff, with increasing burnout of the workforce who have had little opportunity for respite between waves. Low staffing levels are a major risk to patient outcomes [26,27], and very high rates of post-traumatic stress and major depressive disorders have been found amongst healthcare staff after COVID-19 [28], which may persist for several years [29]. This study therefore highlights how strategies to improve wellbeing and optimise staff health and retention are now paramount, such as those published by the American Society of Clinical Oncology [30]. However, each organisation must identify and contextualise their own staff needs and implement evidence-based policies to address these. This must go alongside workforce planning to ensure sufficient training and recruitment. The BGCS workforce survey has demonstrated the number of consultant gynaecological oncologists per-million UK residents varies from 1.25 to 5.0, with a current shortfall of 40 posts (22% of current requirements) [31]. This shortfall is further compounded by the ongoing staff shortages highlighted. This results in persistent and additional capacity constraints in gynaecological cancer care pathways and detrimentally impacts the timely delivery of care, adversely affecting the ability of gynaecological cancer services to not only manage the current workload but also address the backlog from COVID-19. Service providers must urgently consider increasing staffing to allow for recovery of services, resilience to future waves, and retention of a highly trained workforce facing high levels of stress and burnout [23,32]. High quality training with strong pastoral support must be prioritised in order to meet this challenge, with mitigation strategies instituted given the interruption to training seen during these surveys from reduced surgical capacity, reallocation, and sickness.

Gynaecological oncology services must also work to ensure resilience against future epidemics of infectious diseases, and to provide catch-up care to those affected. A key adaptation seen was the relocation of cancer services to ‘COVID-19-free’ sites, which enabled some treatments to continue. These pathways were shown to be associated with reduced morbidity in an international cohort study [33], and hence should be considered as the standard-of-care in the future.

The reduction in suspected cancer referrals during the initial wave [34] has led to early evidence emerging of a resultant stage-shift in cancer presentation [35], although whether this is seen in gynaecological cancers over 2021–2022 remains to be determined. This highlights the pressure likely to be faced by gynaecological cancer services over the coming years. There are major justifiable concerns that the necessary changes in care could result in a detrimental impact on patient outcomes, and guidelines have been published by the BGCS to help mitigate this with salvage therapy or close monitoring of those who received non-standard care [36]. It is also possible that deferral or modification of treatments had no or minimal impact on patients’ outcomes. The ongoing UKCOGS study aims to capture data for all patients discussed at MDT meetings in the UK, identify where therapies were impacted, and assess short and long-term outcomes. UKCOGS has collected data on changes to MDT decision making and the resultant management of over 13,000 UK gynaecological cancer MDT cases during 2020–2021. These analyses should hopefully assist in identifying how to ensure the ‘best care’ in future surges of the COVID-19 pandemic and other similar events.

## 5. Conclusions

This study demonstrates the significant disruption that occurred in gynaecological cancer care in the UK during the three waves of COVID-19, including staff shortages, reduction in urgent referrals, MDT workloads, theatre capacity, and changes to working practices. Whilst disruption eased and referrals/workloads returned to normal over time, significant staff shortages remained in 2022, highlighting the persistent capacity constraints that need urgent redress. Our study offers insights into the adaptations needed to address future pandemics and deal with the resultant backlog from COVID-19.

## Figures and Tables

**Table 1:** Reduction in staff in hospitals.

Hospitals with Significant Staffing Reduction	All			Units			Centres		
Survey 1	25/51 (49%)			8/19 (42%)			17/32 (53%)		
Survey 2	7/42 (17%)			0/13 (0%)			7/29 (24%)		
Survey 3	17/39 (44%)			5/9 (56%)			12/30 (40%)		
Staff Category	Number of Hospitals Reporting Staff Reduction Due to COVID-19- Related Sickness ^1^	Number of Hospitals Reporting Staff Reduction Due to Redeployment ^2^	Proportion of Staff Reduction in Affected Hospitals (%) ^3^	Number of Hospitals Reporting Staff Reduction Due to COVID-19-Related Sickness ^1^	Number of Hospitals Reporting Staff Reduction Due to Redeployment ^2^	Proportion of Staff Reduction in Affected Hospitals (%)^3^	Number of Hospitals Reporting Staff Reduction Due to COVID-19-Related Sickness ^1^	Number of Hospitals Reporting Staff Reduction Due to Redeployment ^2^	Proportion of Staff Reduction in Affected hospitals (%) ^3^
Non-subspecialty trainee doctor staff numbers									
Survey 1	14/25 (56%)	14/25 (56%)	40% (25–100) [1]	5/8 (62%)	4/8 (50%)	30% (25–40) [1]	9/17 (53%)	10/17 (59%)	45% (24–100) [0]
Survey 2	4/7 (57%)	4/7 (57%)	25% (5–75%)				4/7 (57%)	4/7 (57%)	25% (5–75%)
Survey 3	15/39 (38%)	2/39 (5%)	25% (5–35%)	5/9 (56%)	1/9 (11%)	25% (25–35%)	10/30 (33%)	1/30 (3%)	30% (5–30%)
Gynaecological oncology subspecialty trainee numbers (hospitals where applicable)									
Survey 1	2/25 (8%)	6/25 (24%)	100% (100–100) [0]	0/8 (0%)	1/8 (12%)	30% (30–30) [0]	2/17 (12%)	5/17 (29%)	100% (100–100) [0]
Survey 2	1/7 (14%)	0/7 (0%)	45%				1/7 (14%)	0/7 (0%)	45%
Survey 3	7/39 (18%)	1/39 (3%)	0% (0–35%)	3/9 (33%)	1/9 (11%)	15% (8–50%)	4/30 (13%)	0/30 (0%)	0% (0–15%)
Consultant staff numbers									
Survey 1	17/25 (68%)	2/25 (8%)	28% (20–38) [1]	6/8 (75%)	0/8 (0%)	30% (30–33) [1]	11/17 (65%)	2/17 (12%)	25% (20–50) [0]
Survey 2	5/7 (71%)	0/7 (0%)	5% (5–25%)				5/7 (71%)	0/7 (0%)	5% (5–25%)
Survey 3	9/39 (23%)	1/39 (3%)	15% (3–30%)	4/9 (44%)	0/9 (0%)	15% (5–15%)	5/30 (17%)	1/30 (3%)	10% (3–30%)
Clinical nurse specialist staff numbers									
Survey 1	9/25 (36%)	14/25 (56%)	30% (20–50) [2]	4/8 (50%)	6/8 (75%)	40% (20–50) [2]	5/17 (29%)	8/17 (47%)	30% (20–50) [0]
Survey 2	3/7 (43%)	3/7 (43%)	35% (25–45%)				3/7 (43%)	3/7 (43%)	35% (25–45%)
Survey 3	9/39 (23%)	1/39 (3%)	15% (0–30%)	4/9 (44%)	0/9 (0%)	50% (20–80%)	5/30 (17%)	1/30 (3%)	10% (0–30%)

Denominators = number of hospitals. ^1^ Number of hospitals reporting a staffing reduction due to COVID-19-related sickness, out of all hospitals reporting any staffing reduction (%). ^2^ Number of hospitals reporting a staffing reduction due to redeployment, out of all hospitals reporting any staffing reduction (%). ^3^ The proportion by which staffing levels were reduced by, amongst hospitals reporting any staffing reduction: median (Interquartile range) [number of hospitals unknown].

**Table 2:** Structural changes and workload across hospitals.

	Survey 1			Survey 2			Survey 3		
	All	Unit	Centre	All	Unit	Centre	All	Unit	Centre
(a) MDT meeting functioning									
Change to MDT functioning	51/51 (100%)	19/19 (100%)	32/32 (100%)	35/42 (83%)	10/13 (77%)	25/29 (87%)	33/39 (85%)	6/9 (66%)	27/30 (90%)
Moved to virtual meetings	20/51 (39%)	5/19 (26%)	15/32 (47%)	16/42 (38%)	6/13 (46%)	10/29 (34%)	17/39 (44%)	5/9 (56%)	12/30 (40%)
Mixed virtual/F2F meetings	33/51 (65%)	13/19 (68%)	20/32 (62%)	21/42 (50%)	5/13 (38%)	16/29 (55%)	18/39 (46%)	1/9 (11%)	17/30 (57%)
Reduced meeting frequency	0/51 (0%)	0/19 (0%)	0/32 (0%)						
Meetings Suspended	0/51 (0%)	0/19 (0%)	0/32 (0%)						
Meetings Less Attended	21/51 (41%)	6/19 (32%)	15/32 (47%)	7/42 (17%)	2/13 (15%)	5/29 (17%)	5/39 (13%)	1/9 (11%)	4/30 (13%)
% reduction *	40% (17–50) [2]	50% (16–52) [1]	25% (20–40) [1]	25% (5–35)	25% (25–25)	25% (5–60)	25% (25–25)	25% (25–25)	25% (25–40)
% of remote consultations *	75% (50–88) [2]	75% (50–85) [0]	74% (50–87) [2]	25% (5–35) [1]	15% (5–35)	25% (10–45) [1]	0% (0–25)	0% (0–15)	0% (0–25)
(b) Capacity reductions in services *									
Theatre time	40% (20–70) [6]	60% (32–88) [1]	30% (12–55)	10% (0–25)	15% (0–35)	5% (0–25)	0% (0–5)	0% (0–30)	0% (0–0)
Surgical cases postponed	30% (16–57) [9]	35% (16–72) [3]	30% (16–50) [6]	5% (0–15)	5% (5–15)	5%(0–15)	5% (0–5)	5% (5–15)	5% (0–5)
Medical-oncology	0% (0–0) [28]	0% (0–0) [9]	0% (0–13) [19]	0% (0–5) [7]	5% (0–25) [2]	0% (0–5)	0% (0–5) [4]	0% (0–3) [2]	0% (0–5) [2]
Clinical-oncology	0% (0–8) [27]	0% (0–0) [8]	0% (0–10) [19]	0% (0–5) [8]	0% (0–5) [2]	0% (0–5) [6]	0% (0–5) [3]	3% (0–5) [1]	0% (0–3) [2]
Radiology	0% (0–10) [19]	0% (0–0) [6]	0% (0–18) [13]	0% (0–5) [4]	3% (0–5) [1]	0% (0–5) [3]	0% (0–10) [4]	3% (0–15) [1]	0% (0–10) [3]
Pathology	0% (0–0) [15]	0% (0–0) [4]	0% (0–0) [11]	0% (0–5) [2]	0% (0–5)	0% (0–5) [2]	0% (0–15) [2]	0% (0–15)	0% (0–30) [2]
Palliative care	0% (0–0) [28]	0% (0–0) [11]	0% (0–0) [17]	0% (0–5) [1]	0% (0–5)	0% (0–0) [1]	0% (0–0) [4]	0% (0–3)	0% (0–0) [4]
Urgent referrals	50% (25–70) [10]	45% (18–62) [3]	50% (30–70) [7]	0% (0–15) [2]	0% (0–5%)	0% (0–15) [2]	0% (0–3) [3]	0% (0–3)	0% (0–10%) [3]
Weekly MDT meeting list	22% (2–48)	20% (0–30) [1]	28% (14–50) [4]	0% (0–0)	0% (0–0)	0% (0–3%)	0% (0–0)	0% (0–0)	0% (0–0)
(c) Move of activity off-site (another hospital)									
Moved operation lists	23/41 (56%)	9/19 (47%)	14/22 (64%)	17/42 (40%)	5/13 (38%)	12/29 (41%)	4/39 (10%)	1/9 (11%)	3/30 (10%)
Moved clinic	6/40 (15%)	2/19 (11%)	4/21 (19%)	2/42 (5%)	1/13 (8%)	1/29 (3%)	1/39 (3%)	0/9 (0%)	1/30 (3%)
Not yet moved	3/29 (10%)	2/16 (12%)	1/13 (8%)	0/32 (0%)	0/6 (0%)	0/26 (0%)	1/39 (3%)	1/9 (11%)	0/30 (0%)
Central hub for surgical cases	15/39 (38%)	5/18 (28%)	10/21 (48%)	14/37 (38%)	5/10 (50%)	9/27 (33%)	5/39 (13%)	1/9 (11%)	4/30 (13%)
MAS undertaken	30/41 (73%)	11/19 (58%)	19/22 (86%)	41/41 (100%)	13/13 (100%)	28/28 (100%) [1]	39/39 (100%)	9/9 (100%)	30/30 (100%)

* Median (Interquartile range) [number unknown] of the reported % change across different hospitals. MDT: multidisciplinary team. MAS: minimal access surgery.

## Data Availability

Publicly available datasets were analysed in this study. This data and R code used for analysis can be found here: https://github.com/brentnall/paper-ukcogs-survey (accessed on 31st October 2022).

## Supplementary Materials

The following supporting information can be downloaded at: https://www.mdpi.com/article/10.3390/cancers15041273/s1, Table S1: COVID-19 Survey of Gynae-oncology Centres and Units.
