# Supplementary material for: Impact of Multiple COVID-19 Waves on Gynaecological Cancer Services in the UK
Source: Cancers (Basel). 2023 Feb 16;15(4):1273. doi: 10.3390/cancers15041273 (PMC9953843; doi:10.3390/cancers15041273)
Supplement: Supplementary file 1 [file cancers-15-01273-s001.zip › cancers-2114294-supplementary.pdf]

Table S1: COVID-19 Survey of Gynae-oncology Centres and Units

**Do you represent a Centre or Unit**	**Centre ☐**	**Unit ☐**	
Name of Centre or Unit			
**Regarding staffing, have you experienced significant reduction in staff numbers**	**Yes ☐**	**No ☐**	
**If Yes, is this due to COVID related sickness or redeployment or both (please enter/tick accordingly). For example if Junior doctor staff numbers are reduced from 10 to 7 (i.e. by 30%) then enter 30 in the % reduced column.**	**COVID**	**Redeployment**	**% reduced**
Junior doctor staff numbers	☐	☐	
GO Sub-specialty Trainee numbers (hospitals where applicable)	☐	☐	
Consultant staff numbers	☐	☐	
CNS staff numbers	☐	☐	
**Regarding MDT functioning, have you implemented any changes**	Yes ☐	No ☐	
If Yes, tick all that apply			
Moved to Virtual MDT	☐		
Mixed virtual and face to face MDT	☐		
Suspended MDT	☐		
Reduced MDT frequency	☐		
Reduced number of attendees	☐	% reduced ______	
**Regarding Out-patient clinics**			
What proportion of your out-patient clinic is remote consultation (telephone/ video)			______
**Please answer questions below in terms of Gynae Oncology related activity: (for example if number of theatre sessions are reduced from 10 to 7 (i.e. by 30%) then enter 30 in the % reduced column.**			**% reduced**
Re theatre time, how much reduction in theatre time has occurred? (If not reduced enter '0')			________
What is the proportion of surgical cases postponed (if no cases postponed, enter '0')			________
Re Medical Oncology access/capacity, how much is this reduced by? (If not reduced enter '0', If unknown enter NK)			________
Re Clinical Oncology access/capacity, how much is this reduced by? (If not reduced enter '0', If unknown enter NK)			________
Re Radiology access/capacity, how much is this reduced by? (If not reduced enter '0', If unknown enter NK)			________
Re Pathology access/capacity, how much is this reduced by? (If not reduced enter '0', If unknown enter NK)			________
Re Palliative care access/capacity, how much is this reduced by? (If not reduced enter '0', If unknown enter NK)			________
How much have your rapid access referrals dropped by? (If not reduced enter '0')			________
How much has your weekly MDT list/workload reduced by? (If not reduced enter '0')			________
**Have you needed to move activity off site to another hospital (e.g. Independent sector)**	Yes	No	
Moved operating lists	☐	☐	
Moved Clinics	☐	☐	
Moved other activity	☐	☐	
Not yet moved but are planning to move	☐	☐	
Do you need to go via a central hub or committee to access operating	☐	☐	
**Are you undertaking Minimal access procedures**	Yes ☐	No ☐	
**Please use the box below to provide any free text comments.**			
